# Sucralose Influences the Productive Performance, Carcass Traits, Blood Components, and Gut Microflora Using 16S rRNA Sequencing of Growing APRI-Line Rabbits

**DOI:** 10.3390/ani14131925

**Published:** 2024-06-29

**Authors:** Hatem M. El-Tahan, Mohamad Elsayed Elmasry, H. A. Madian, Ahmad R. Alhimaidi, In Ho Kim, Jae Hong Park, Hossam M. El-Tahan

**Affiliations:** 1 Animal Production Research Institute (APRI), Agricultural Research Center (ARC), Ministry of Agriculture, Dokki, Giza 12611, Egypt; hatem_eltahan2002@yahoo.com (H.M.E.-T.); melmasry93@arc.sci.eg (M.E.E.); heshammadian2@gmail.com (H.A.M.); 2 Postdoc at the Department of Animal Science, Jeonbuk National University, Jeonju 54896, Republic of Korea; 3 Department of Zoology, College of Science, King Saud University, Riyadh 11451, Saudi Arabia; 4 Animal Resource and Science Department, Dankook University, Cheonan 31116, Republic of Korea; 5 Smart Animal Bio Institute, Dankook University, Cheonan 31116, Republic of Korea

**Keywords:** rabbits, prebiotic, sucralose, blood parameters, microbial activity, growth performance

## Abstract

**Simple Summary:**

This study investigated the impact of sucralose on rabbit intestine and caecal microbial activity, as well as various physiological parameters and performance indicators. One hundred and sixty 5-week-old rabbits were divided into four groups and administered different doses of sucralose. The results showed improved weight gain and feed conversion ratios in rabbits given sucralose, without significant effects on mortality. Sucralose altered blood parameters, decreasing glucose and triglyceride levels while increasing total lipids and cholesterol. It also influenced gut microbiota, increasing beneficial bacteria and decreasing harmful ones. The study suggests that caution should be taken in using sucralose, as it may have both positive and negative effects on rabbit health and gut microbiota.

**Abstract:**

This study investigated how sucralose influenced rabbit intestine and caecal microbial activity, blood parameters, growth performance, carcass characteristics, and digestibility. In total, 160 5-week-old rabbits from the APRI line weighing 563.29 gm were randomly assigned to four experimental groups with four replicates—5 males and 5 females in each. Four experimental groups were used, as follows: SUC1, SUC2, and SUC3 got 75, 150, and 300 mg of sucralose/kg body weight in water daily, while the control group ate a basal diet without supplements. The results showed that both the control and SUC1 groups significantly (*p* < 0.05) increased daily weight gain and final body weight. Sucralose addition significantly improved feed conversion ratio (*p* < 0.05) and decreased daily feed intake (gm/d). The experimental groups do not significantly differ in terms of mortality. Furthermore, nutrient digestibility was not significantly affected by sucralose treatment, with the exception of crud protein digestion, which was significantly reduced (*p* < 0.05). Additionally, without altering liver or kidney function, sucralose administration dramatically (*p* < 0.05) decreased blood serum glucose and triglyceride levels while increasing total lipids, cholesterol, and malonaldehyde in comparison to the control group. Furthermore, the addition of sucrose resulted in a significant (*p* < 0.05) increase in the count of total bacteria, *lactobacillus*, and *Clostridium* spp., and a decrease in the count of *Escherichia coli*. Further analysis using 16S rRNA data revealed that sucralose upregulated the expression of *lactobacillus* genes but not that of *Clostridium* or *E. Coli* bacteria (*p* < 0.05). Therefore, it could be concluded that sucralose supplementation for rabbits modifies gut microbiota and boosts beneficial bacteria and feed conversion ratios without side effects. Moreover, sucralose could decrease blood glucose and intensify hypercholesterolemia and should be used with caution for human consumption.

## 1. Introduction

The increasing demand for poultry meat has led to the intensive use of antibiotics in the rabbit industry to relieve the challenges of weaning stress, including decreased feed intake, stunted growth, and elevated mortality rates [1]. However, as a consequence of this approach, bacterial strains that are resistant to antibiotics have developed, threatening human and animal health. Therefore, alternative strategies for increasing feed intake and controlling pathogenic bacteria in the rabbit gut are urgently needed to reduce the weaning stress.

Sucralose is a non-nutritive sweetener frequently used as a sugar substitute in various foods and beverages since sucralose is 600 times sweeter than sugar. Berry et al. [2] and Magnuson et al. [3] observed its safety without creating toxicity in several studies on mice, rats, rabbits, and dogs. It has been reported that most of the ingested sucralose cannot be broken down in the body [4] and eliminated from the body without giving any caloric output [2]. Prebiotics are indigestible food components that selectively stimulate one or several household bacteria to affect the host positively [5]. Moreover, the diverse community of bacteria known as the gut microbiome lives in the gastrointestinal tract and is engaged in some metabolic functions, such as immune system regulation, digestion, and the synthesis of vital vitamins and nutrients. It has been proven by several studies that sucralose succeeds in alternating the growth of bacteria in the human oral cavity [6], human gut [7,8,9], and mice gut [10,11,12], as well as environmental microbiota [13,14]. However, there is no report about the impact of chronic exposure to sucralose on the diversity of gut bacteria in rabbits. Furthermore, it has been observed that oral sucralose causes hyperphagia in mice and fruit flies by stimulating central NPY functions [15,16]. In addition, Zhang et al. [17] illustrated that 150 mg/kg sucralose raised feed intake and boosted the weaned piglets’ growth performance. The APRI-line rabbits from the Animal Production Research Institute in Egypt are a new rabbit line with high production efficiency which were adapted to Egyptian environmental conditions via mating between *Baladi red* bucks and Spanish *V-Line* does, resulting in F1, F2, and F3 generations, with selection beginning at this generation for hot environmental resistance and mothering ability [18]. Their well-characterized genetic makeup, adaptability, and resilience make them an ideal choice for investigating the impacts of dietary additives and nutritional strategies on rabbit health, welfare, and performance. This study hypothesises that the sucralose addition to the feed mixture will positively affect rabbit performance during weaning period. Therefore, the current study intends to investigate the effect of sucralose on rabbit growth performance, digestibility, blood parameters, carcass features, and gut microbiome populations. These findings may have crucial ramifications for creating novel dietary interventions that benefit human and animal health.

## 2. Material and Methods

### 2.1. Animals

This study was conducted on the rabbit farm at Sakha Station, Animal Production Research Institute (APRI), Agriculture Research Centre (ARC), Egypt. Throughout the experiment, the animals (aged 5 to 13 weeks) were provided with unlimited access to food and water. Every five rabbits from the replicates were placed into the wire-meshed cages (50 × 50 × 40 cm) from 5 to 9 weeks of age and then were housed individually in metal wire-meshed cages (50 × 35 × 35 cm) under identical management circumstances from 9 to 13 weeks of age at a constant temperature of 25 ± 1 °C and humidity of approximately 60%. The study followed the instructions of the animal committee at the Animal Production Research Institute under the ethical approval number 2023-0302APRI.

### 2.2. Experimental Design

For this experiment, 160 5-week-old rabbits with an average weight off 563.29 gm were randomly assigned to four experimental groups with four replicates, each consisting of 5 males and 5 females with similar body weights (BW). Four experimental groups were used: control, SUC1, SUC2, and SUC3 received 0, 75, 150, and 300 mg of sucralose/kg BW/day, respectively, added to their water, according to Eltahan et al. [19] and Del Pozo et al. [14]. The dose was 20 times higher than the acceptable daily intake (ADI) dose from the US Food and Drug Administration, which was 15 mg/kg BW/day for humans. It should be noted that there may be people whose consumption exceeds the ADI dose and some of the short-term interventions with sucralose in amounts below the ADI found no significant effect among the groups [7]. Data were recorded regarding the quantity of deceased rabbits, live weight, amount of feed consumed, and amount of water consumed. Sucralose (C12H19Cl3O8) with CAS number (56038-13-2), product number (PHR1342) and a quality level of 300 was purchased from Sigma-Aldrich Co., St. Louis, Mo., USA. The nutritional composition and formulation of the basal diet calculated according to De Blas and Mateos [20], which were created to satisfy all of the rabbits’ growing needs for nutrients, are shown in Table 1.

### 2.3. Data Collection

The diet was provided twice per day. The rest of the feedstuff in the feeder was gathered and weighed, and the average feed intake for each weekly interval in replications was calculated to determine the amount of feed consumed. Water consumption for each group was measured by recorded the initial water levels in each group of rabbit’s water container at the beginning of the experiment and weighed again the next morning before refilling it to determine the daily water intake by subtract the daily final water level from the initial water level. Average weekly measurements of body weight were performed, and the feed conversion rate, death rate, daily weight gain, and group-level performance indexes were also calculated. Moreover, the performance index was calculated as follows:

The final live body weight (kg) divided by the feed conversion ratio (%) multiplied by 100.

### 2.4. The Carcass Traits and Serum Blood Analysis

Six 13-week-old rabbits (3 males and 3 females) were randomly chosen from each treatment after the growth period, starved for 12 h, weighed, and euthanized by isoflurane anaesthesia (Mylan Inc., Tokyo, Japan) before being slaughtered to estimate some of the carcass features [21,22]. Parts of the carcass were displayed as a percentage of live body weight. Blood samples from six rabbits for each treatment group were obtained to investigate certain blood constituents. Using commercial kits (Bio-Diagonosis Co., Cairo, Egypt), the following procedures were followed to determine blood serum total protein, albumin, cholesterol, triglycerides, glucose, ALT (alanine aminotransferase), AST (aspartate aminotransferase), urea, and creatinine by colorimeter. The difference between albumin and total protein was used to obtain globulin values.

### 2.5. Digestibility and Cecum Bacteriology Experiment

Twenty male APRI-line rabbits with similar body weights took part in a digestibility trial after the growth experiment to assess the apparent nutrient digestibility of the four experimental diets (five males in each). The metabolic cages used to keep the animals allowed to separate urine and faces individually. The daily faces were gathered into polyethylene bags and kept at a temperature of −20 °C for five consecutive days [23] in compliance with the European reference technique for rabbit digestion studies. Following AOAC [24], chemical analyses of meals, hard faces, and meat samples were performed for CP, ash, DM, EE, and CF. Under Mackie and McCartney [25], the American Public Health Association, APHA [26], and the Difco Manual [27], the microbiological identification investigation was performed on samples of caecum contents from the carcass traits rabbit (six rabbits in each).

### 2.6. Isolation of Gut Bacteria DNA and qPCR Analysis Based on 16s rRNA Gene

The six cecum samples (200 mg) from each group as above mentioned were used to extract bacterial DNA by using the bead-beating approach, which was then purified, as previously mentioned by Matsuki et al. [28], with some modifications. Briefly, 200 μL of the faecal sample diluents were added to 1 mL of phosphate buffer solution (PBS) once the sample had thawed, and the mixture was thoroughly vortexed. Centrifugation at 20,000× *g* for 5 min at 4 °C was followed by a discard of the supernatant and two washes with 1 mL of PBS solution to eliminate PCR inhibitors. 400 μL of supernatant and 400 microliter of phenol, chloroform, and isoamyl alcohol (25:24:1; *v*/*v*) were combined, and the mixture was well mixed with a Micro Smash, running at 4800 rpm for 45 s. Following a 5 min centrifugation at 20,000× *g* at 4 °C, 250 μL of supernatant and 25 μL of 3 M sodium acetate (pH 5.2) were combined. Following a 3-min ice-cold storage period, 300 μL of ice-cold 100% isopropanol was added, and the mixture was centrifuged at 20,000× *g* for 5 min at 4 °C. After being cleaned with 500 μL of ice-cold 70% ethanol, the DNA pellet was allowed to air dry and then suspended in 1 mL of Tris-EDTA buffer (pH 8.0), then kept at −30 °C until needed. The bacterial DNA from the cecum was examined for the expression of *lactobacillus*, *Escherichia coli*, and *Clostridium perfringens* by using real-time PCR according to the procedures previously described in Eltahan et al. [29]. Table 2 lists the primer sequences. Contamination handled via UV irradiation and negative controls for all the steps.

The PCR-ct results against all-bacteria primerwas normalized by Furet et al. [30] who demonstrated that primer could be used to detect all bacteria. Then, the results for *Lactobacillus*, *Escherichia coli*, and *Clostridium perfringens* were computed using the 2^−ΔΔCt^ method as mentioned in Bahry et al. [33] and Eltahan et al. [34]. The specificity of the PCR settings was verified by identifying the single melting signal that was within the standard curves for every sample, and that was an outlier that did not undergo further analysis.

### 2.7. Statistical Analysis

A one-way ANOVA was used to conduct the statistical analysis and the General Linear Model Program of SAS [35] on data related to growth performance, digestibility, blood, caecal microbial activity, and carcass features. Multiple range tests were performed by Duncan [36] in order to identify statistically significant differences between means. To remove outliers (*p* < 0.01), all data were subjected to a Thompson’s rejection test, as explained by Kobayashi and Pillai [37]. The findings are shown as mean values with combined standard errors (SEM). The results were deemed significant at *p* < 0.05.

## 3. Results

### 3.1. Growth Performance

The impact of sucralose on growth performance from 5 to 13 weeks of age is displayed in Table 3. No significant variation in the initial body weight has been found at five weeks of age. Significant variations occurred between 6 and 13 weeks when the growing period ended. The rabbits supplemented with SUC2 and SUC3 had the lowest final body weight, while the rabbits treated with 75 mg of sucralose and those that remained untreated had the highest final body weight. During the initial phase (5–9 weeks of age), there was a significant decline in daily weight gain as the amount of sucralose administration increased. However, during the second period, no significant modifications were seen (9–13 weeks of age).

Throughout the entire trial (5–13 weeks of age), the daily weight gain of the rabbits was significantly higher value by the SUC1 and control groups, while the SUC2 and SUC3 treatments demonstrated a significantly lower value (26.46 vs. 26.84 gm, *p* < 0.001). Feed intake was significantly (*p* < 0.001) decreased overall during the period (5–13 weeks of age) when sucralose levels were raised; the same pattern was observed in the first and second periods. Regarding feed conversion, no significant variations between treatments could be seen during the initial phase (5–9 weeks of age). The rabbits from the SUC3 group had the best feed conversion ratio throughout the second period (9–13 weeks of age), while the rabbits given the control had the worst values. Throughout the entire time, the same trend was observed. Moreover, sucralose showed no significant effect on the amount of water consumed. The rabbit from control and SUC1 groups had the highest performance index % (*p* < 0.05) compared to other groups. There was no significant variation in the mortality rate across the experimental groups. Nonetheless, the rabbits that got SUC3 treatments had the lowest mortality compared to the rest of the groups.

### 3.2. Carcass Traits and Blood Components

The carcass yield affected by sucralose is represented in Table 3. Total edible represents dressing-out percentage and significantly declined (*p* < 0.001) with rising levels of sucralose in water. Gastrointestinal tract percentage was increased (*p* < 0.001) as the levels of sucralose increased in water. 

The results concerning blood components are listed in Table 4. There was no significant effect in the serum total protein of the rabbits who received sucralose supplementation in water compared with the control group. The glucose and triglyceride significantly declined while cholesterol, total lipids, low-density lipoprotein (LDL), high-density lipoprotein (HDL), and malonaldehyde significantly increased sucralose dose-dependently compared to the control.

### 3.3. The Digestibility Traits and Caecum Microbial Activity

The nutritional digestibility data are displayed in Table 5. The digestibility of nutrients was not significantly affected by adding sucralose to rabbit water, except for the digestion coefficient of CP, which was significantly reduced. 

The impact of sucralose addition on caecum microbial activity in rabbits represented in Table 6. Raising the amounts of sucralose in rabbit water caused a significant (*p* < 0.001) decrease in the ammonia contents of the caecum. When compared to control treats, the caecum content of rabbits given sucralose in water showed a significant rise in the overall count of total bacteria, *lactobacillus*, and *clostridium* spp., while the number of pathogenic bacteria (*Escherichia coli*) significantly decreased.

### 3.4. The qPCR Deferential of Lactobacillus, E. coli, and Cl. Perfringens in the Caecum of Rabbits after Sucralose Administration

**Figure 1:**
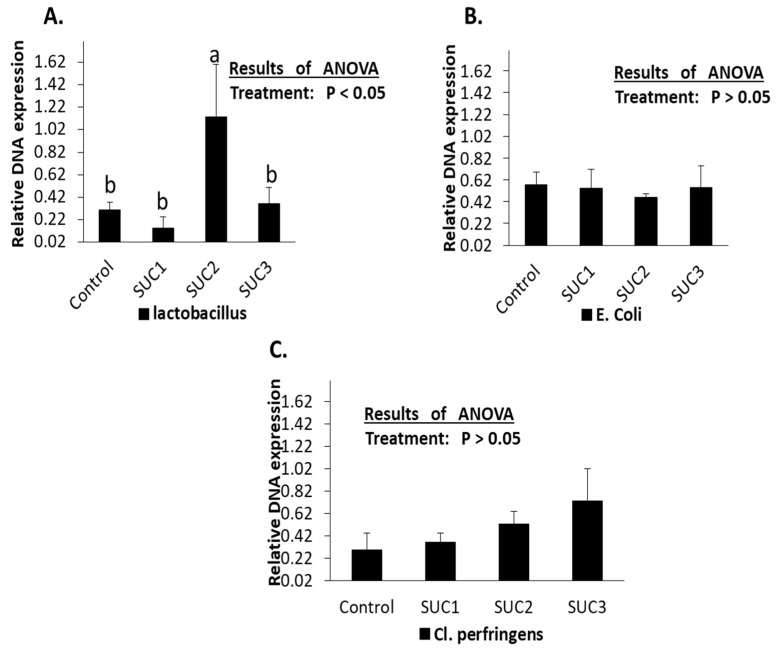
The 16s rRNA of the total amount of *lactobacillus* (**A**), *E. coli* (**B**), and *Cl. perfringens* (**C**) in the cecum of rabbit following administration of sucralose (75, 150, and 300 mg/kg of body weight). ^a,b^ Means on the bar superscript are significantly different (*p* < 0.05); values are mean ± SEM for each group of 6 rabbits in each group.

The microbial activity evaluation using 16S rRNA is stated in Figure 1. The total number of *lactobacillus* in caecum samples significantly increased (*p* < 0.05) after 150 mg sucralose administration. In comparison, the low dose of 75 mg and high dose of 300 mg did not change the expression of *lactobacillus* bacteria (Figure 1A). Additionally, the expression of *E. coli* and *cl. perfringens* bacteria did not show significant (*p* > 0.05) alteration with sucralose administration. (Figure 1B,C). 

## 4. Discussion

This study investigates the impact of sucralose on rabbit growth performance, gut microbial activity, digestibility, carcass features, and blood parameters.

Among the mammals that can feel sweetness are rabbits because their taste buds have highly developed sweet taste receptors [38]. Table 3 showed that adding dose-dependent sucralose to rabbit water significantly (*p* < 0.05) reduced feed intake, daily weight gain, and final body weight. This study’s findings were similar to those noted by Goldsmith [39] and Kille et al. [40], who discovered that rats’ body weights and food intakes were decreased by large dosages of sucralose. However, Eltahan et al. [19] showed that body weight and feed intake in chicks were unaffected by sucralose oral injection. Furthermore, Soffritti et al. [41] found that feeding mice sucralose at 0, 500, 2000, 8000, and 16,000 ppm did not affect food intake or body weight. Throughout the trial (5–13 weeks of age), there were significant variations (*p* < 0.001) in the feed conversion ratio. The rabbits that got 150 mg/kg sucralose in water had the best values, while those fed a control diet had the lowest. The drop in feed intake could be the result of sweetness taste from sucralose supplementation to the rabbit’s water.

Moreover, sucralose had no significant effect on water consumption. The SUC1 group had the highest performance index percentage (*p* < 0.05), which could be attributed to a combination of factors, including an increase in ultimate body weight and a decrease in feed conversion ratio. There was no significant variation in the mortality among the groups that were also obtained by Goldsmith [39], who found that there were no deaths among the treatment groups in which male and female rats were given sucralose at doses of 0, 750, 1500, or 3000 mg/kg/day for 26 weeks. Dressing out percentage represent as total edible significantly declined (*p* < 0.05) dose-dependently with sucralose, as shown in Table 3. This reduction may be due to decreased DM intake with the abundant sucralose treatments. The gastrointestinal tract percentage increased as the levels of sucralose increased in water. The higher percentage of the gastrointestinal tract may be due to the carcass’s lower dressing content, which can be explained by Goldsmith [39], who found that sucralose dose-dependently increased the weight of the caecal tissue in rats. 

In Table 4, sucralose administration had no significant impact on blood total protein value, AST and ALT as liver function enzymes, or urea and creatinine as kidney function compared with the control group. The serum glucose and triglyceride significantly declined (*p* < 0.001) with increasing sucralose levels in water, while sucralose administration significantly dose-dependently increased serum cholesterol and total lipids. These findings stand with Saada et al. [42], who found that sucralose oral administration of 11 mg/kg for six weeks decreased the glucose and triglyceride levels while increasing cholesterol, LDL, and HDL in diabetic rats. Malonaldehyde, as an end product for free radicals, works as a biomarker for oxidative stress [43]. Sucralose dose-dependently (*p* < 0.05) increased the malonaldehyde level in serum compared to the control. That means sucralose has an oxidative stress effect. This result is similar to Erbaş et al. [44], who illustrated that long-term consumption of artificial sweeteners (sucralose) impairs memory learning and raises oxidative stress levels in rats. On contrast, Jiang et al. [45] found that the oxidative state and MDA concentration in the broiler chicken jejunal mucosa were unaffected (*p* > 0.05) by sweetener supplementation. 

Sucralose administration showed no (*p* > 0.05) effect on nutrient digestibility, Table 5, except for the digestion coefficient of CP, which significantly declined with provided sucralose. This study’s results are similar to those of Goldsmith [39], who reported that high concentrations of sucralose reduced palatability and digestibility in rats. Moreover, Schiffman et al. [46] recently revealed that sucralose administration causes leaky gut syndrome and poor digestion in an in vitro study that may explain the poor CP digestibility in this study.

Concerning the effect of sucralose on gut bacteria, chronic oral sucralose consumption has been reported to induce alteration in the gut bacteria dynamic system in mice [47], which is consistent with the current study, Table 6. We found that sucralose significantly (*p* < 0.05) increased the cecum total number of bacteria, *lactobacillus*, and *clostridium* spp. Meanwhile, the total number of E. coli significantly declined when sucralose was added to rabbit water. Moreover, the 16S rRNA technique in this study (Figure 1) provided more evidence for the rabbit gut alteration; hence, we found that Suc2 (150 mg/kg of sucralose) increased (*p* < 0.05) cecum total number of *lactobacillus* (Figure 1A) while *E. coli* and *Cl. Perfringens* did not significantly change among the groups (*p* > 0.05) in cecum gut samples (Figure 1B,C), which is not in line with previous studies that indicating the inhibition effect of sucralose on the bacteria [6,13,48], which might be due to the age of rabbits now allowing the stability of gut microbiota or the short exposure period of sucralose. According to Abou-Donie et al. [48] study, sucralose negatively impacted gut flora in rats orally splenda gavage at 100, 300, 500, or 1000 mg/kg doses for 12 weeks. These findings demonstrated that sucralose consumption causes an imbalance in the gut microbiota—notably, a reduction in the total number of anaerobic and aerobic bacteria—as well as a notable decline in the number of advantageous anaerobic bacteria (for instance, *Bacteroides*, *lactobacilli*, and *bifidobacteria*). 

Similarly, Uebanson et al. [49] suggested that sucralose intake dose-dependently affected the relative proportion of *clostridium* spp. in mice. Moreover, the increment of *clostridia* spp in this study can be explained by Suez et al. [10], who found that the consumption of sucralose could alter the gut microbiota composition and their function, hence raising the risk of glucose intolerance and that related to an increment in *clostridial* and *Bacteroides* spp. after fecal transplants in germ-free mice. In addition, the number of *E. coli* declined in this study, and the same results were obtained by Corder and Knobbe [50], who found that sucralose suppressed the growth of the control *E. coli* strain. Shil and Chichger [51] found that sucralose did not succeed (*p* < 0.05) in changing the growth of *E. coli*. The current study stated that microbiota distribution in the rabbit cecum may change if sucralose is added to the water. Numerous studies have demonstrated the same sucralose alteration trend on gut bacteria; Sánchez-Tapia et al. [52] examined the possible differences in the impact of sweetener type and high-fat diet on gut flora control. It was stated that sucralose increased the abundance of firmicutes while *Bacteroides* showed a declining tendency, lowering alpha diversity. Additionally, Wang et al. [53] conducted an 8-week study on mice administered sucralose. They noticed an increase in the Firmicutes group’s abundance but no changes in alpha diversity, Actinobacteria, or Proteobacteria.

Furthermore, artificial sweeteners, including saccharin, sucralose, and aspartame, have been demonstrated to negatively affect intestinal permeability and epithelial cell apoptosis, which raises the possibility that bacteria will penetrate the gut epithelium and cause septicemia [50]. Generally, the production of neuropeptides and short-chain fatty acids by the microbiota in the gut is significant and may impact animals’ feed consumption [54]. Due to their insensitivity to sweetness, sucralose supplementation-induced inhibition of feed intake may be linked to the release of anorexigenic neuropeptides from the hypothalamus or the gut microbiome. This hypothesis must be verified and looked into for further investigation.

## 5. Conclusions

Sucralose administration in rabbit water reduces body weight, feed intake, and CP digestibility, while sucralose enhances the feed conversion ratio. Sucralose modifies the body’s metabolism to induce hyperlipidemia upon hypoglycemia. Sucralose raises oxidative stress by increasing the level of malonaldehyde. Sucralose altered the gut microbiota, increasing the total bacteria, *lactobacillus*, and *clostridium* counts while reducing the number of *E. coli* bacteria. Overall, additional investigation is required to understand how sucralose affects gut flora; current data indicates that sucralose may have unexpected effects and should be used cautiously for human. Those who want to cut less sugar can use molasses, honey, and maple syrup as natural sweeteners.

## Figures and Tables

**Table 1:** Composition and chemical analysis of basal diet.

Ingredients	%	Chemical Analysis (% as DM):	%
Berseem hay	30.05	Dry matter (DM)	85.81
Barley grain	24.60	Crude protein (CP)	17.36
Wheat brain	21.50	Organic matter	91.42
Soybean meal (44% CP)	17.50	Crude fibre	12.37
Molasses	3.00	Ether extract	2.230
Limestone	0.95	Digestible energy (MJ/kg DM)	10.11
Di-calcium phosphate	1.60	Calcium	1.243
Sodium chloride	0.30	Phosphorus	0.808
Mineral–vitamin premix ^(1)^	0.30	Methionine	0.454
DL-Methionine	0.20	Lysine	0.862
		Nitrogen-free extract (NFE)	56.7
		Ash 8.6 ADF 16.25	
		NDF 28.75 ADL 3.1	
Total	100		

^(1)^ Premix provided per kg of diet: 12,000 IU of vitamin A; 2500 IU of vitamin D3; 40 mg of vitamin E; 2.0 mg of vitamin K; 2.0 mg of vitamin B1; 4 mg of vitamin B2; 2.0 mg of vitamin B6; 0.01 mg of vitamin B12; 0.06 mg of biotin; 50 mg of niacin; 0.3 mg of folic acid; 10 mg of D-pantothenic acid; 1000 mg of choline; 40 mg of Zn; 10 mg of Cu; 30 mg of Mn; 50 mg of Fe; 0.5 mg of I; 0.2 mg of Se; 0.5 mg of Co. NDF, neutral detergent fibre (%); ADF, % acid detergent fibre in DM; ADL, acid detergent lignin.

**Table 2:** Primers used for real-time PCR based on 16s rRNA gene for bacteria.

Gene	Accession No.	Sequences 5′−3′ (Forward/Reverse)	Annealing Temperature (°C)	Product Size (bp)	Reference
All bacteria ^#^	_	5′−CGGTGAATACGTTCCCGG−3′/ 5′−TACGGCTACCTTGTTACGACTT−3′	60	145	Furet et al. [30]
*Cl. perfringens*	NR_121697.2	5′−AAGATGGCATCATCATTCAACCA−3′/ 5′−GTGCAATATTCCCCACTGCTGCCT-3′	60	188	This study
*E. coli*	J01859.1	5′−AAGACCAAAGAGGGGGACCT−3′/ 5′−TGTCTCAGTTCCAGTGTGGC−3′	60	141	Kramski et al., [31]
*Lactobacillus* *	NC_004957.1	5′−GGCAGAGCCTCCATAAGCAA−3′/ 5′−GACGATTTTTCGTCTCGGCG−3′	60	278	This study

All bacteria (^#^) primers have been used to determine all of the bacteria species in the samples via real-time PCR according to Furet et al. [30]. Primers were designed with Primer-Blast (http://www.ncbi.nlm.nih.gov/tools/primer-blast/); *Cl. perfringens* accessed on 20 January 2006, *E. coli* was accessed on 1 June 1972, and *Lactobacillus* accessed on 15 July 2001. All bacteria and *lactobacillus* primer were aligned with the program Clustal W (Thompson et al. [32] using the 16s rRNA gene. (*) Modified from the reference.

**Table 3:** Effect of sucralose supplementation level on growth performance of growing APRI-line rabbits.

Parameters	Control	SUC1	SUC2	SUC3	SEM	*p* Value	Linear *p* Value	Quadratic *p* Value
No. of rabbits at 5 weeks of age	20	20	20	20	-	-	-	-
Final number at 13 weeks of age	19	19	19	18	-	-	-	-
Initial body weight (g)	581.2	581.2	582.0	580.0	6.078	0.89	0.855	0.8401
Final body weight (g)	2158.2 ^A^	2132.7 ^A^	2084.7 ^B^	2061.5 ^B^	12.28	0.007	0.00455	0.2301
Daily weight gain (g):								
5–9 weeks	28.80 ^A^	27.82 ^AB^	27.04 ^BC^	26.38 ^C^	0.488	0.009	0.0680	0.802
9–13 weeks	27.42	27.64	26.63	26.54	0.624	0.41	0.8.80	0.4301
5–13 weeks	28.11 ^A^	27.73 ^A^	26.84 ^B^	26.46 ^B^	0.218	0.005	4.80 × 10^5^	0.2012
Feed intake (g/d):								
5–9 weeks	80.08 ^A^	74.06 ^B^	73.22 ^BC^	71.16 ^C^	0.703	0.009	2.80 × 10^7^	1 × 10^3^
9–13 weeks	113.5 ^A^	106.1 ^B^	104.7 ^B^	102.6 ^B^	1.173	0.007	6.80 × 10^5^	0.0802
5–13 weeks	96.78 ^A^	90.07 ^B^	88.96 ^BC^	86.89 ^C^	0.874	0.005	5.80 × 10^6^	4 × 10^3^
Feed conversion ratio:								
5–9 weeks	2.806	2.675	2.725	2.703	0.060	0.07	0.693	0.4329
9–13 weeks	4.184 ^A^	3.876 ^B^	3.958 ^AB^	3.894 ^B^	0.080	0.031	0.0393	0.1054
5–13 weeks	3.442 ^A^	3.250 ^B^	3.317 ^B^	3.288 ^B^	0.027	0.006	2.93 × 10^6^	0.2019
Water consumption (mL/d):								
5–9 weeks	132.6	131.2	130.7	129.8	2.108	0.42	0.4980	0.9571
9–13 weeks	211.2	210.4	208.4	207.2	2.383	0.76	0.1745	0.9427
5–13 weeks	171.9	170.8	169.6	168.5	1.654	0.41	0.2110	0.9851
Performance index (%)	62.77 ^B^	65.75 ^A^	62.97 ^B^	62.82 ^B^	0.642	0.045	0.0593	0.2149
Total edible (%) BW	59.02 ^A^	56.84 ^B^	56.41 ^B^	55.97 ^B^	0.438	0.008	0.00455	0.148061
GIT ^(1)^ (%) BW	12.64 ^C^	13.93 ^B^	14.03 ^AB^	14.09 ^AB^	0.324	0.004	0.0293	0.1229
Mortality rate (%) ^(2)^	5	5	5	10	-	-	-	-

SUC1: rabbit group received 75 mg/kg BW sucralose in water; SUC2: rabbit group received 150 mg/kg BW sucralose in water; SUC3: rabbit group received 300 mg/kg BW sucralose in water; SEM = standard error of means; ^A,B,C^ means in the same row with different superscripts are significantly different (*p* < 0.05); ^(1)^ GIT: gastrointestinal tract; ^(2)^ chi-square test.

**Table 4:** Effect of sucralose supplementation level on some blood parameters of growing APRI-line rabbits.

Parameter	Control	SUC1	SUC2	SUC3	SEM	*p*-Value	Linear *p* Value	Quadratic *p* Value
Total protein (g/dL)	6.08	5.92	5.86	5.80	0.145	0.15	0.184	0.72605
Glucose (mg/dL)	89.33 ^A^	80.00 ^B^	77.73 ^B^	68.33 ^C^	1.919	0.004	9.27 × 10^7^	0.98128
Cholesterol (mg/dL)	69.95 ^B^	71.88 ^B^	73.95 ^A^	76.38 ^A^	0.831	0.007	2.75 × 10^5^	0.72519
H.D.L. (mg/dL)	33.15 ^B^	34.22 ^A^	34.84 ^A^	35.09 ^A^	0.184	0.004	4.08 × 10^4^	0.15381
L.D.L. (mg/dL)	30.15 ^C^	30.69 ^BC^	31.12 ^AB^	31.75 ^A^	0.136	0.008	7.81 × 10^4^	0.63631
Total lipids (g/L)	294.0 ^C^	301.7 ^BC^	309.3 ^B^	323.0 ^A^	3.804	0.006	4.41 × 10^5^	0.35780
Triglyceride (mg/dL)	90.05 ^A^	88.09 ^B^	86.44 ^C^	85.12 ^D^	0.614	0.006	4.60 × 10^5^	0.58383
Creatinine (mg/dL)	0.95	0.91	0.91	0.92	0.016	0.08	0.274	0.07555
Urea (mg/dL)	15.25	15.10	14.95	14.91	0.207	0.09	0.00347	0.12595
AST (U/L)	22.91	22.39	22.70	22.91	0.721	0.74	0.921	0.62929
ALT (U/L)	15.59	14.69	14.71	15.17	0.693	0.85	0.724	0.40206
Malonaldehyde (mg/mL)	1.108 ^C^	1.155 ^C^	1.284 ^B^	1.415 ^A^	0.028	0.009	1.03 × 10^4^	0.29308

SUC1: rabbit group received 75 mg/kg BW sucralose in water; SUC2: rabbit group received 150 mg/kg BW sucralose in water; SUC3: rabbit group received 300 mg/kg BW sucralose in water; SEM = standard error of means; ^A,B,C,D^ means in the same row with different superscripts are significantly different (*p* < 0.05).

**Table 5:** Effect of sucralose supplementation level on apparent digestibility (%) of growing APRI-line rabbits.

Nutrient	Control	SUC1	SUC2	SUC3	SEM	*p*-Value	Linear *p* Value	Quadratic *p* Value
Dry matter (DM)	71.15	71.16	71.38	71.50	0.443	0.75	0.409	0.878
Organic matter (OM)	66.09	66.56	66.72	66.75	0.704	0.45	0.519	0.774
Crude protein (CP)	75.68 ^A^	74.36 ^BC^	75.22 ^AB^	73.78 ^C^	0.316	0.054	0.0280	0.902
Crude fibre (CF)	30.38	30.69	30.94	31.08	0.491	0.42	0.662	0.946
Ether extract (EE)	78.55	78.11	77.81	77.63	0.652	0.86	0.578	0.916
Nitrogen-free extract (NFE)	51.47	52.29	52.87	53.22	0.714	0.12	0.255	0.837

SUC1: rabbit group received 75 mg/kg BW sucralose in water; SUC2: rabbit group received 150 mg/kg BW sucralose in water; SUC3: rabbit group received 300 mg/kg BW sucralose in water; SEM = standard error of means; ^A,B,C^ means in the same row with different superscripts are significantly different (*p* < 0.05).

**Table 6:** Effect of sucralose supplementation level on microbial activity of growing APRI-line rabbits.

Parameters	Control	SUC1	SUC2	SUC3	SEM	*p*-Value	Linear *p* Value	Quadratic *p* Value
No. of rabbits	4	4	4	4	-	-		
pH	6.72	6.52	6.32	6.35	0.104	0.95	0.0599	0.458
NH_3_ (mmol/L)	8.20 ^A^	5.35 ^B^	3.22 ^C^	3.32 ^C^	0.234	0.007	9.79 × 10^6^	3.19 × 10^3^
Total bacterial count (×10^6^) ^(1)^	12.2 ^C^	16.12 ^B^	24.85 ^A^	23.75 ^A^	1.862	0.04	9.06 × 10^7^	0.0259
*Lactobacilli* (×10^5^) ^(1)^	2.25 ^D^	14.45 ^C^	20.25 ^A^	16.45 ^B^	0.371	0.004	2.32 × 10^4^	2.71 × 10^9^
*Escherichia coli* (×10^4^) ^(1)^	12.92 ^A^	7.42 ^B^	3.28 ^C^	3.42 ^C^	0.649	0.007	3.77 × 10^7^	1.04 × 10^6^
*Clostridium* spp. ^(1)^	2.12 ^C^	4.42 ^B^	2.52 ^C^	7.35 ^A^	0.406	0.008	1.86 × 10^3^	0.120

SUC1: rabbit group received 75 mg/kg BW sucralose in water; SUC2: rabbit group received 150 mg/kg BW sucralose in water; SUC3: rabbit group received 300 mg/kg BW sucralose in water; SEM = standard error of means; ^A,B,C,D^ means in the same row with different superscripts are significantly different (*p* < 0.05). ^(1)^ Germ counts expressed in CFU/g caecal digesta.

## Data Availability

The authors confirm that the data supporting the findings of this study are available upon the request from the corresponding author.
